# Relationship between Pulse Pressure and Handgrip Strength in the Korean Population: A Nationwide Cross-Sectional Study

**DOI:** 10.3390/jcm13051515

**Published:** 2024-03-06

**Authors:** Ryuk Jun Kwon, Young Hye Cho, Eun-Ju Park, Youngin Lee, Sang Yeoup Lee, Jung-In Choi, Sae Rom Lee, Soo Min Son

**Affiliations:** 1 Family Medicine Clinic and Research Institute for Convergence of Biomedical Science and Technology, Pusan National University Yangsan Hospital, Yangsan 50612, Republic of Korea; brain6@hanmail.net (R.J.K.);; 2 Department of Family Medicine, Pusan National University School of Medicine, Yangsan 50612, Republic of Korea; 3 Department of Medical Education, Pusan National University School of Medicine, Yangsan 50612, Republic of Korea

**Keywords:** pulse pressure, sarcopenia, cardiovascular disease, muscular strength, prevalence

## Abstract

**Background:** Sarcopenia is defined as the loss of muscle mass and strength and low physical performance, and it is closely related to the risk of cardiovascular disease and mortality. Pulse pressure (PP) is a biomarker of arterial stiffness and compliance. Elevated PP levels increase the risk of cardiovascular diseases and all-cause mortality. Nevertheless, the association between PP and sarcopenia has not yet been clearly established. **Methods:** Participant data were extracted from the Korea National Health and Nutrition Examination Survey conducted from 2014 to 2020. The study population was classified into three groups (PP < 40 mmHg, 40 mmHg ≤ PP < 60 mmHg, and PP ≥ 60 mmHg). PP was calculated by deducting the diastolic blood pressure from the systolic blood pressure. For handgrip strength, the maximum value measured with a grip dynamometer was adopted (weak handgrip strength: <28 kg for men, <18 kg for woman; normal handgrip strength: ≥28 kg for men, ≥18 kg for women). To determine the relationship between PP and the prevalence of weak handgrip strength, multiple logistic regression analysis was performed after adjusting for possible confounding factors. **Results:** The higher PP group had a higher age, body mass index; systolic blood pressure, prevalence of hypertension, diabetes, hyperlipidemia, and metabolic syndrome, and maximum handgrip strength. In all models, the prevalence of weak handgrip strength was significantly higher in the group with PP ≥ 60 mmHg compared to the control group (PP < 40 mmHg). **Conclusions:** Elevated PP was significantly associated with a higher prevalence of weak muscle strength. Thus, PP monitoring may be used to identify individuals at risk of sarcopenia and is helpful in improving health outcomes.

## 1. Introduction

Sarcopenia is the age-associated loss of muscle mass and strength and physical function [1,2]. Sarcopenia is closely associated with falls, fractures, physical activity, cardiovascular disease, and mortality [3,4,5,6]. Although the prevalence of sarcopenia may vary significantly depending on the classification criteria and chosen cutoff thresholds, it is 8–36% for individuals under 60 years and 10–27% for those over 60 years [7]. According to population projections until 2045, the estimated European population with sarcopenia will increase from 10,869,527 in 2016 to 18,735,173 in 2045, reflecting a 72.4% increase, suggesting that sarcopenia will be a public health problem over the next 30 years [8]. Therefore, recognizing the risk factors of sarcopenia and its indicators is essential for early diagnosis or the implementation of targeted interventions. This is important for preventing or managing a range of adverse health outcomes, regardless of whether an individual has comorbidities.

Pulse pressure (PP) represents the difference between systolic and diastolic blood pressure, and it is closely related to arterial stiffness, cardiac output, and wave reflection [9]. The correlation between PP and arterial stiffness is strong, as age-related vascular calcification and elastin degradation contribute to arterial stiffness, leading to increased PP [10]. Elevated PP raises the likelihood of developing chronic kidney disease, cardiovascular disease, and all-cause mortality [11,12,13]. However, the relationship between PP and handgrip strength (HGS), an important factor in sarcopenia, has not been thoroughly explored.

The purpose of the present study is to determine if PP and HGS are associated, using data from the Korea National Health and Nutrition Examination Survey (KNHANES). To do this, the relationship between PP and clinical characteristics was examined, and changes in PP and the prevalence of weak HGS according to age and sex were identified. Finally, the correlation between PP and the prevalence of weak HGS was assessed.

## 2. Materials and Methods

### 2.1. Study Population

This study was conducted using data from the 2014–2020 KNHANES, which are data composed of a complex sampling design including variance estimation, stratification variables, and sample weights. The Korea Disease Control and Prevention Agency conducts this cross-sectional population-based survey annually. These tests include health examinations, health interviews, and nutrition surveys collected through standardized physical examinations, questionnaires, blood/urine tests, and radiologic tests. Because this is a public survey, the data were obtained from https://knhanes.kdca.go.kr/knhanes/main.do, accessed on 11 February 2024. Among the 54,668 participants, this study did not include the following: (a) participants under the age of 19 years; (b) those diagnosed with any type of cancer, including stomach, liver, colon, breast, lung, cervix, thyroid, and other cancers; (c) participants with angina pectoris or myocardial infarction at baseline; (d) participants diagnosed with stroke from the beginning; and (e) those with missing data. The present study was approved by the Institutional Review Board of Pusan National University Yangsan Hospital (IRB No. 04-2023-027).

### 2.2. Study Variables

Clinical factors, including body mass index (BMI, kg/m^2^), systolic blood pressure (SBP, mmHg), and diastolic blood pressure (DBP, mmHg), were extracted from the physical examinations. Information regarding additional factors, such as smoking status, alcohol intake, physical activity, hypertension, diabetes, hyperlipidemia, and metabolic syndrome, was obtained from health interviews. Smokers (current or previous smokers) and never smokers were the two categories of smoking status. Participants who drank more than once a month in the previous year were considered drinkers. Regular physical activity was defined as high-intensity exercise for at least 1.5 h per week, moderate-intensity exercise for at least 2.5 h per week, or a combination of both (1 min of high intensity was equivalent to 2 min of moderate intensity).

Participants who met the criteria for hypertension (SBP ≥ 140 mmHg or DBP ≥ 90 mmHg), or who were taking antihypertensive medications, were classified as having hypertension. If a participant’s fasting glucose level was >126 mg/dL or they were receiving treatment for diabetes, they were considered to have diabetes mellitus. According to the KNHANES definition, participants were deemed to have hyperlipidemia if their total cholesterol levels increased by more than 240 mg/dL or if they were prescribed medication for hyperlipidemia. Metabolic syndrome is characterized by the existence of three or more of the following features: (1) waist circumference exceeding 90 cm for men or 85 cm for women, (2) high blood pressure (SBP ≥ 135 mmHg or DBP ≥ 85 mmHg) or use of medication for hypertension, (3) elevated triglyceride in serum over 150 mg/dL or medication for hyperlipidemia, (4) low level of high-density lipoprotein cholesterol (<40 mg/dL for men, <50 mg/dL for women), (5) high fasting blood glucose levels exceeding 100 mg/dL or use of medication for diabetes.

### 2.3. Pulse Pressure

PP was calculated as the difference between SBP and DBP. Blood pressure was checked by four trained nurses in charge of measuring blood pressure at the Korea Disease Control and Prevention Agency. The average of each participant’s second and third blood pressure measurement was chosen after each was performed three times. If the arm is not at the standard level when taking blood pressure, it should be adjusted for hydrostatic pressure and with the suggested addition or subtraction of 0.7 mmHg for vertical height either above or below the heart level, according to the American Heart Association [14]. Therefore, the measurement of blood pressure was adjusted using the average arm height (83 cm for men and 81 cm for women) that corresponded to its height. The participants were divided into three groups according to the PP interval (PP < 40 mmHg, 40 mmHg ≤ PP < 60 mmHg, PP ≥ 60 mmHg).

### 2.4. Handgrip Strength

The HGS test was performed using a digital grip strength dynamometer (T.K.K 5401, Tokyo, Japan). The participants were directed by trained technicians to maintain a seated position, ensuring their distal interphalangeal finger joints formed a 90-degree angle to the handle. They were then instructed to exert maximum pressure by squeezing the dynamometer. In 2014–2017, the measurement method started with the dominant hand, and both hands were crossed and measured three times. From 2018, the maximum value was measured for the grip strength of both hands or one hand, and these were also measured three times. The maximum value was obtained as the HGS. The participants were divided into weak (<28 kg for men and <18 kg for women) and normal HGS (≥28 kg for men, ≥18 kg for women) groups based on the 2019 Asian Working Group for Sarcopenia [2].

### 2.5. Statistical Analyses

To identify representative samples at the national level, a complex sampling design that included integrated weights, stratification, and clusters was employed, along with the suggested guidelines for data analysis from the KNHANES.

Analysis of variance and Chi-squared tests were performed to evaluate the baseline characteristics of the participants. Data are presented as mean ± standard error for continuous variables, or as numbers and weighted percentages for categorical variables. To determine the relationship between PP and HGS, a multiple logistic regression analysis was conducted after adjusting for possible confounders: (a) age, (b) sex, (c) BMI, (d) history of smoking status, (e) current drinking status, (f) physical activity, (g) hypertension, (h) diabetes mellitus, and (i) hyperlipidemia. The statistical analyses were carried out using the IBM Statistical Package for the Social Sciences version 21 (United States). Statistical significance was set at *p* < 0.05.

## 3. Results

### 3.1. Baseline Characteristics of Study Population

**Figure 1:**
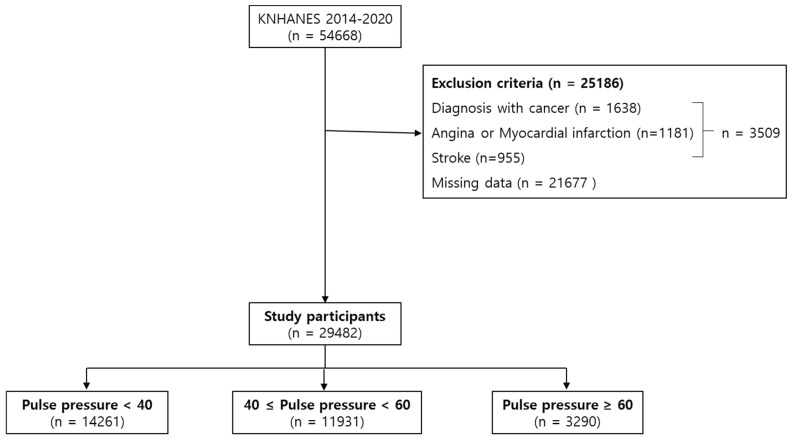
Flow diagram of study participants in KNHANES 2014–2020. KNHANES: Korea National Health and Nutrition Examination Survey; n: number. A total of 3509 individuals with diagnoses of cancer, angina, or myocardial infarction, and stroke at baseline, were recorded.

A total of 54,668 individuals initially participated in the study. Participants diagnosed with cancer, angina pectoris, myocardial infarction, or stroke, as well as those younger than 19 years old or with missing information, were excluded, and then 29,482 participants remained. Among them, there were 14,261 participants with a PP less than 40 mmHg, 11,931 with a PP between 40 and 60 mmHg, and 3290 with a PP of 60 mmHg or more (Figure 1).

Table 1 presents the clinical characteristics of the participants at baseline. The mean age, sex, and BMI were significantly different among the PP groups. The PP of 60 mmHg or higher group had the oldest participants, followed by a PP of 40–60 mmHg, and the lowest average age group had a PP of less than 40 mmHg. In addition, the BMI tended to elevate as the difference in PP increased. However, there was no trend in the percentage of sex by PP group. SBP showed a significant improvement as the difference in PP increased, whereas DBP showed a significant difference by PP group; however, there was no tendency due to an increase in PP. The smaller the difference in PP, the higher the proportion of participants who regularly practiced physical activity and the higher the proportion in terms of alcohol consumption. On the other hand, in terms of smoking status, there was a significant difference between the PP groups; however, no significant trend was observed. Moreover, the proportion of patients with hypertension, diabetes, hyperlipidemia, and metabolic syndrome, which affect vascular stiffness, was significantly higher in the group with a larger PP difference.

### 3.2. The Value of HGS according to PP Interval

**Figure 2:**
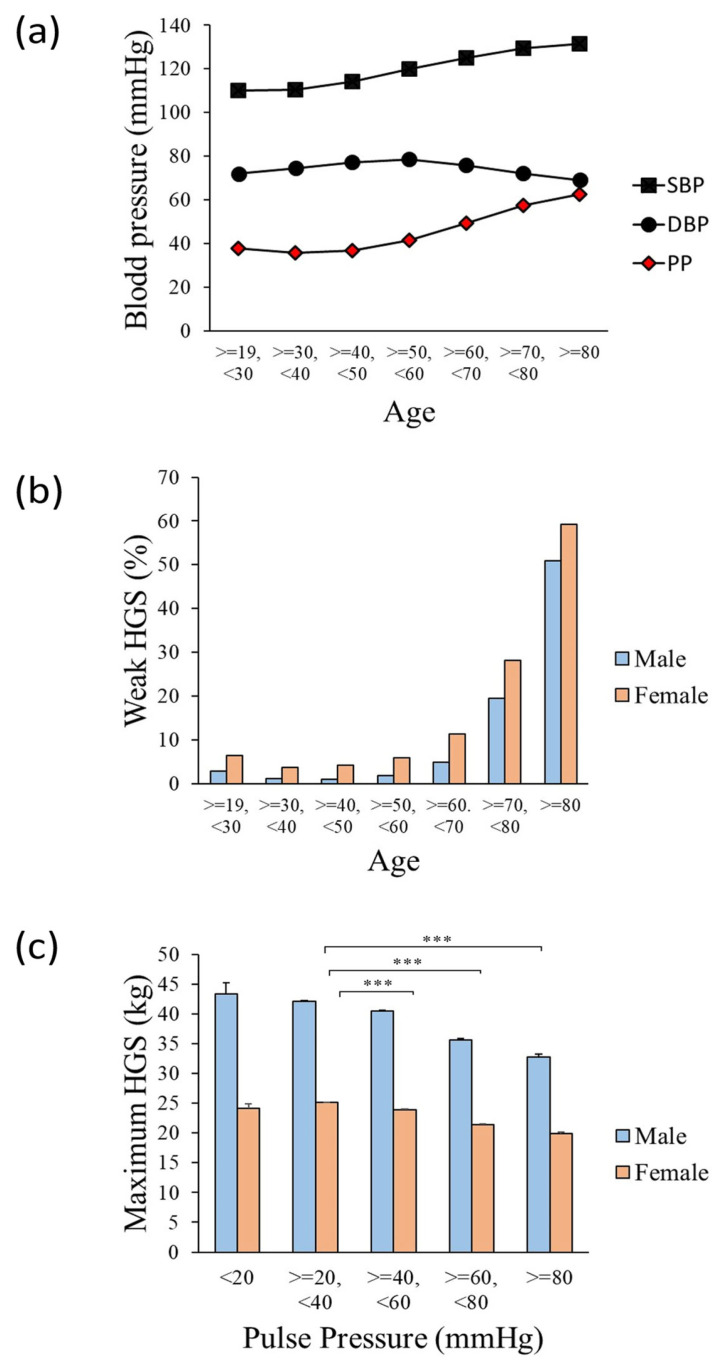
Tendency of blood pressure and prevalence of weak HGS by age, and maximum HGS by PP intervals. (**a**) Changes in SBP, DBP, and PP according to age. SBP (blue), DBP (orange), PP (gray). (**b**) Prevalence of weak HGS in males and females among different age groups. Male (blue), female (orange). (**c**) Average maximum HGS in males and females among different PP intervals. Reference group (20 ≤ PP < 40 mmHg), *t*-test, *** *p* < 0.001, male (blue), female (orange). DBP: diastolic blood pressure; HGS: handgrip strength: PP, pulse pressure; SBP: systolic blood pressure.

To evaluate the significance of PP in skeletal muscle strength, changes in the prevalence of weak HGS and PP according to age were evaluated (Figure 2). SBP tended to increase with age, although DBP tended to increase in youths and decrease after the age of 50–60 years (Figure 2a). After 50 years of age, the PP value increased to 40 mmHg or more and had a propensity to increase with age. Moreover, the percentage of participants with weak HGS increased with age in both males and females (Figure 2b). In addition, the maximum HGS was evaluated as PP increased in both males and females. When compared to the PP ≥ 20, <40 mmHg group, the maximum HGS decreased significantly as PP increased in both men and women (Figure 2c).

### 3.3. Relationship between PP and the Prevalence of HGS

A multiple logistic regression analysis was performed (Table 2) to evaluate the association between PP and HGS. Compared to participants in the PP less than 40 mmHg group, those in the 40 ≤ PP < 60 mmHg and PP ≥ 60 mmHg groups had a significantly higher prevalence of weak HGS, and the PP ≥ 60 mmHg group had the highest prevalence of weak HGS (Model 1; 40 ≤ PP < 60 mmHg: odds ratio 1.639, confidence interval [CI] 1.440–1.865/PP ≥ 60 mmHg: odds ratio 6.346, CI 5.529–7.284). Next, because differences in the prevalence of HGS occurred with increasing age or depending on sex, they were adjusted for (Model 2). In the age- and sex-adjusted model, the odds ratio for the prevalence of weak HGS was 1.550 in the PP ≥ 60 mmHg group compared to that in the reference, but there was no significant difference in the 40 ≤ PP < 60 mmHg group compared to that in the reference (Model 2; 40 ≤ PP < 60 mmHg: odds ratio 0.950, CI 0.840–1.075/PP ≥ 60 mmHg: odds ratio 1.550, CI 1.325–1.815). After adjusting for potential confounding variables, PP ≥ 60 mmHg was significantly correlated with a higher prevalence of weak HGS (Model 3; PP ≥ 60 mmHg: odds ratio 1.582, CI 1.291–1.937).

## 4. Discussion

In the present study, we evaluated the correlation between PP and patient characteristics, and identified the correlation between PP and HGS in Korean adults using KNHANES data.

High PP was correlated with hypertension, diabetes, hyperlipidemia, and metabolic syndrome, because these factors play an important role in arterial stiffness and cardiovascular disease [15,16,17,18,19]. Elevated PP, a sign of increased arterial stiffness, was more frequently observed in patients with cardiovascular disease and hypertension [15,16] and was a risk factor for diabetic mellitus in females [19]. Moreover, PP and metabolic syndrome were positively correlated in Korean and European adults [17,18]. Similarly, in the present study, the higher PP group had a higher proportion of patients diagnosed with hypertension, diabetes, and hyperlipidemia (Table 1). These findings suggest that high PP is associated with an increased risk of metabolic and cardiovascular diseases.

The PP and prevalence of low HGS increase with age [20,21,22]. An observational study showed that PP in males increased after their fifties, while that in females increased after their forties [22]. Abe et al. revealed that HGS was similar among younger age groups (20 to 49 years old) and gradually decreased with increasing age [21]. Consistent with this research, the present study demonstrated an age-associated positive correlation between PP and the prevalence of weak HGS in the Korean population (Figure 2). These findings demonstrate the significance of PP and HGS as key age markers. In addition, the association between PP and HGS implies that elevated PP could play a potential role in sarcopenia, leading to increased arterial stiffness and reduced arterial compliance.

Age-related decreases in muscle strength, mass, and function are referred to as sarcopenia, which can result in reduced physical performance and an increased risk of adverse health outcomes. Among these, HGS, which indicates skeletal muscle strength [2], is a key factor of sarcopenia and is used as a biomarker, which is consistent in explaining bone mineral density, fractures, quality of life, diabetes, arterial stiffness, cardiovascular disease, and all-cause mortality [23,24,25,26,27,28,29,30,31,32,33,34]. Dixon et al. showed that weak grip strength is associated with an increased risk of vertebral fracture and poor bone mineral density in the hips and spine in women [25]. According to a systematic review, all 11 included studies showed that HGS is associated with an increased hip fracture risk [26]. In addition, both HGS and quality of life were positively correlated with old age, advanced cancer, and chronic obstructive pulmonary disease [27,28,29]. A study revealed that grip strength was lower in adults (aged ≥ 20 years) with diabetes with no history of cardiovascular disease [30]. Several studies have shown that HGS is negatively associated with arterial stiffness in non-hypertensive participants, patients with diabetes, and patients with chronic coronary syndrome [31,32,33]. Moreover, weak grip strength was associated with a higher risk of mortality from cardiovascular disease, respiratory disease, and cancer in a prospective cohort study of half a million UK Biobank participants [34].

High PP, which is strongly correlated with arterial stiffness and predicts atherosclerosis-related complications, is also positively correlated with coronary heart disease, heart failure, overall cardiovascular events, and cardiovascular mortality [10,35,36]. Although both PP and sarcopenia are associated with cardiovascular disease, few studies have yet confirmed the relationship between PP and HGS [37,38]. It has been found that in comparison to older women without sarcopenia, high PP in those with sarcopenia was related to poor muscle function and high cardiovascular risk [37]. Ohara et al. found a significant negative correlation between PP and HGS in 1593 middle-aged to older participants without a history of symptomatic cardiovascular events such as coronary heart disease, peripheral arterial disease, stroke, or congestive heart failure [38]. Consistent with these findings, high PP was associated with weak HGS in the Korean population (Table 2). Therefore, high PP may be a risk factor for sarcopenia.

Comprehensively, the association between PP and HGS indicates that PP may be a useful functional and prognostic variable in low muscle strength. Early detection of increased PP may allow for intervention at the right time, including the management of malnutrition and exercise, to prevent sarcopenia and improve overall health. Ischemic preconditioning, which stands as a proven technique for organ adaptation to chronic ischemia, is known to enhance peripheral blood circulation [39]. For example, walking training, one of the ischemic preconditioning methods, may be used as a way to slow down or prevent sarcopenia.

### 4.1. Possible Mechanism

Several possible pathways have been considered, although the precise processes related to PP and sarcopenia are poorly understood. First, because high PP indicates hard arterial stiffness and low arterial compliance, this may result in endothelial dysfunction by reducing microcirculation, which may lead to muscle fiber atrophy and sarcopenia [40,41]. Second, arterial stiffness, which is closely related to oxidative stress and chronic molecular inflammation, plays an important role in muscle inflammation and loss [40,41]. The presence of inflammatory factors and reactive oxygen species is associated with a potential inhibition of protein synthesis and an increase in protein degradation, potentially contributing to the development of sarcopenia [40]. Nevertheless, further studies are required to determine the mechanisms by which high PP can result in sarcopenia and to identify promising treatment targets for the avoidance of sarcopenia related to arterial stiffness.

### 4.2. Limitations and Strengths

The present study has several limitations. First, the causal relationship between PP and HGS could not be clarified because this was a cross-sectional study. Second, because information regarding the type of antihypertensive agents, anticoagulants, and inotropic drugs known to affect PP was not available [42], this study was not adjusted for them. However, this study reflects the diversity present in the general population of South Korea because participant data in the KNHANES cover a wide range of demographic factors, such as age, gender, and geographic location. To the best of our knowledge, this is the first study to determine the relationship between PP and HGS in the Korean population.

### 4.3. Future Directions of Studies

Muscle strength is influenced not only by PP but also by aging, poor nutrition, lack of exercise, frailty syndrome [43,44,45], and hormone deficiency. Therefore, a longitudinal study is required to determine the causal relationship of muscle strength caused by PP. In addition, examining physiological pathways, such as arterial stiffness and endothelial function, will be able to deepen our comprehension of the association between these two parameters.

## 5. Conclusions

The results of this study support the hypothesis that an elevated PP is positively correlated with a higher prevalence of sarcopenia, which can be inferred from a weak HGS. These findings highlight the significance of high PP as a possible risk factor for sarcopenia. Additionally, PP monitoring may be able to serve as a convenient screening approach to identify individuals prone to developing sarcopenia. Detecting and intervening early, particularly targeting arterial stiffness, may alleviate the burden of sarcopenia and enhance overall health outcomes among the Korean population. Future studies should concentrate on clarifying the connections between PP and sarcopenia, seeking to reveal the underlying mechanisms, because this exploration is crucial for identifying innovative therapeutic targets and interventions in the prevention and treatment of sarcopenia.

## Figures and Tables

**Table 1:** Baseline characteristics of study participants.

	N	PP < 40 (*n* = 14,261)	40 ≤ PP < 60 (*n* = 11,931)	PP ≥ 60 (*n* = 3290)	*p* Value
**Age** (y) **Sex** (male) **BMI** (kg/m^2^) **SBP** (mmHg) **DBP** (mmHg)	29,482 29,482 29,425 29,482 29,482	40.63 ± 0.138 5968 (41.8) 23.40 ± 0.036 108.24 ± 0.111 75.32 ± 0.107	47.98 ± 0.239 5567 (46.7) 24.37 ± 0.041 122.85 ± 0.144 76.38 ± 0.131	66.18 ± 0.363 1260 (38.3) 24.42 ± 0.083 144.46 ± 0.353 74.53 ± 0.316	<0.001 <0.001 <0.001 <0.001 <0.001
**Smoking status**, *n* (%) Smokers Never smokers	29,310 11,481 17,829	5565 (43.1) 8653 (56.8)	4783 (44.3) 7070 (55.7)	1133 (38.0) 2106 (62.0)	<0.001
**Drinking**, *n* (%) (yes)	26,236	8614 (64.7)	6479 (63.4)	1231 (51.0)	<0.001
**Physical activity**, *n* (%) (regular)	29,309	7026 (52.0)	5364 (49.5)	1135 (37.8)	<0.001
**Hypertension**, *n* (%) (yes)	29,482	1831 (11.4)	4160 (27.2)	2724 (75.4)	<0.001
**Diabetes**, *n* (%) (yes)	28,598	786 (5.1)	1542 (10.9)	875 (27.3)	<0.001
**Hyperlipidemia**, *n* (%) (yes) **Metabolic syndrome**, *n* (%) (yes)	28,601 29,475	2109 (13.1) 2602 (16.7)	2858 (19.6) 4096 (27.7)	1034 (31.7) 1853 (52.2)	<0.001 <0.001
**HGS**, *n* (%) (weak)	29,482	670 (6.6)	1071 (11.1)	747 (23.5)	<0.001

*p* < 0.05, conducted by ANOVA or Chi-square test. Values are presented as number (weighted percentage) or mean ± standard error. Abbreviations: ANOVA: analysis of variance; BMI: body mass index; DBP: diastolic blood pressure; HGS: handgrip strength; N: number; PP: pulse pressure; SBP: systolic blood pressure.

**Table 2:** Association between pulse pressure and handgrip strength.

	Model 1	*p* Value	Model 2	*p* Value	Model 3	*p* Value
**Pulse pressure**						
**<40**	Reference		Reference		Reference	
**≥40, <60**	1.639 (1.440–1.865)	<0.001	0.950 (0.840–1.075)	N.S.	0.935 (0.807–1.084)	N.S.
**≥60**	6.346 (5.529–7.284)	<0.001	1.550 (1.325–1.815)	<0.001	1.582 (1.291–1.937)	<0.001

Values are expressed as odds ratio (95% CI); *p* value < 0.05; Model 1 was not adjusted; Model 2 was adjusted for age and sex; Model 3 was adjusted for age, sex, BMI, smoking status, drinking, physical activity, hypertension, diabetes, and hyperlipidemia. Abbreviation: BMI, body mass index; CI, confidence intervals; N.S., not significant.

## Data Availability

KNHANES for use of the data (obtained from https://knhanes.kdca.go.kr/knhanes/main.do, accessed on 11 February 2024).

## Abbreviations

BMI	body mass index
BP	blood pressure
CI	confidence interval
DBP	diastolic blood pressure
HGS	handgrip strength
KNHANES	Korea National Health and Nutrition Examination Survey
PP	pulse pressure
SBP	systolic blood pressure
